# MRI-based radiomics-deep learning model for preoperative pathogen prediction in perianal abscesses

**DOI:** 10.3389/fmed.2026.1865284

**Published:** 2026-06-17

**Authors:** Weiping Lu, Jiajia Wang, Yan Li, Na Jiang, Ying Wang, Bingcang Huang, Wei Xing

**Affiliations:** 1 Department of Radiology, Third Affiliated Hospital of Soochow University, Changzhou, Jiangsu, China; 2 Department of Radiology, Pudong Gongli Hospital, Shanghai University of Medicine and Health Sciences, Shanghai, China; 3 School of Gongli Hospital Medical Technology, University of Shanghai for Science and Technology, Shanghai, China; 4 Department of Traditional Chinese Medicine Anorectal Surgery, Pudong Gongli Hospital, Shanghai University of Medicine and Health Sciences, Shanghai, China; 5 Department of Clinical Laboratory Medicine, Pudong Gongli Hospital, Shanghai University of Medicine and Health Sciences, Shanghai, China; 6 Shanghai Health Commission Key Lab of Artificial Intelligence (AI)-Based Management of Inflammation and Chronic Diseases, Sino-French Cooperative Central Lab, Pudong Gongli Hospital, Shanghai University of Medicine and Health Sciences, Shanghai, China

**Keywords:** deep learning, *Escherichia coli*, magnetic resonance imaging, perianal abscess, radiomics

## Abstract

**Objective:**

This study aimed to develop a hybrid model combining MRI-based radiomics, deep learning, and clinical variables for preoperative differentiation of *Escherichia coli* from non-*Escherichia coli* pathogens in perianal abscesses.

**Methods:**

A retrospective series of 215 patients with culture-confirmed perianal abscesses (119 *Escherichia coli*, 96 non-*Escherichia coli*) was analyzed. Preoperative MRI data were collected, with radiomic and deep learning features extracted from T1-weighted imaging (T1WI), T2-weighted imaging (T2WI), and fat-suppressed T2WI (FS-T2WI) sequences. Radiomic feature selection was performed using univariate *t*-tests, Pearson correlation, and least absolute shrinkage and selection operator (LASSO) regression. Clinical and MRI data were screened through univariate and multivariable logistic regression. The MRI signature was derived by averaging the probabilities from a logistic regression model (radiomics) and a k-nearest neighbors classifier (deep learning). A hybrid logistic regression model integrated the MRI signature with clinical predictors to create a nomogram. Model performance was evaluated using receiver operating characteristic (ROC) analysis, calibration curves, and decision curve analysis (DCA).

**Results:**

*Escherichia coli* accounted for 57.63% of all strains. Gender (odds ratio [OR] = 0.456, *p* = 0.001) and diabetes (OR = 9.207, *p* < 0.001) were significant independent predictors. The nomogram achieved an area under the curve (AUC) of 0.885 in the testing set, outperforming the MRI signature alone (AUC = 0.860), with accuracy of 0.815, sensitivity of 0.818, and specificity of 0.812. Calibration curves showed good agreement between predicted and observed outcomes, while DCA demonstrated superior clinical utility.

**Conclusion:**

The hybrid model, utilizing preoperative multi-sequence MRI, noninvasively identifies *Escherichia coli*, the predominant pathogen in perianal abscesses, offering significant clinical potential to transition from empirical antibiotic regimens to microbiology-guided precision strategies.

## 1 Introduction

Perianal abscess, or anorectal abscess, represents a frequently encountered surgical condition defined by the localized collection of inflammatory fluid in perianal soft tissues or adjacent anatomical compartments. Approximately 90% of cases originate from infections of the anal crypt glands (1). Epidemiological data indicate annual incidences of 68,000–96,000 cases in the United States and 14,000–20,000 in the United Kingdom (1, 2), with a Swedish cohort study reporting a population-based incidence of 16.1 per 100,000 (3). Established risk factors include smoking, elevated body mass index (BMI), suboptimal glycemic control, and inflammatory bowel disease (4–7). The classic clinical triad of pain, localized swelling, and fever typically guides diagnosis (8). Anatomically, perianal abscesses are classified by their spatial relationships, with ischiorectal (also called ischioanal) abscesses being the most common subtype, followed by intersphincteric, supralevator, and submucosal variants (8).

Microbiological profiling reveals dual pathogen reservoirs: gut-derived and skin-associated flora. A meta-analysis of Chinese studies identified *Escherichia coli* (64%), *Klebsiella pneumoniae* (13%), and *Staphylococcus* species (7%) as predominant isolates, predominantly Gram-negative enteric organisms (9). Critically, enteric pathogens demonstrate stronger associations with subsequent fistula formation compared to skin-derived species (10). The heterogeneous antibiotic susceptibility patterns among these pathogens necessitate tailored antimicrobial therapy, particularly in patients with cellulitis, systemic comorbidities, or immunocompromised status (8). Moreover, postoperative antibiotics following incision and drainage of perianal abscess may reduce fistula formation risk (11, 12).

Magnetic resonance imaging (MRI) has become the cornerstone of preoperative evaluation for perianal abscesses and fistulas, endorsed by international guidelines (8, 13). Its non-invasive nature, absence of ionizing radiation, and superior soft tissue contrast—enhanced by phased-array coil technology—enable comprehensive multi-planar assessments. MRI excels in delineating deep-seated collections (e.g., supralevator abscesses) and complex configurations (e.g., horseshoe abscesses) that evade digital rectal examination (14), thereby informing surgical planning and complication monitoring. Advanced MRI sequences, including diffusion-weighted imaging (DWI), magnetic resonance spectroscopy (MRS), and susceptibility-weighted imaging (SWI), have proven valuable in distinguishing pyogenic (bacterial), tuberculous, and fungal brain abscesses (15–17). Nevertheless, the potential of MRI for discriminating microbial etiologies in perianal abscesses remains unexplored.

Timely pathogen identification during perioperative or conservative management is paramount for optimizing antibiotic stewardship, curtailing resistance, and improving outcomes. Current pathogen identification in perianal abscesses depends on intraoperative pus cultures, which are susceptible to multifactorial failure risks, while reliable preoperative microbiological profiling methods remain unavailable. Radiomics extracts sub-visual, quantitative imaging features—including texture patterns and deep learning signatures—to decode lesion heterogeneity and bridge macroscopic imaging phenotypes with molecular-pathological profiles, generating clinically actionable biomarkers for precision diagnosis and targeted therapy (18–20). Leveraging these advances, we hypothesized that radiomic signatures could non-invasively classify perianal abscess pathogens. Our study aimed to develop a hybrid predictive model by integrating traditional radiomics and deep learning models via an averaging ensemble method, combined with clinical baseline data and MRI characteristics, to classify perianal abscess pathogens into *Escherichia coli* and non-*Escherichia coli* categories using preoperative MRI.

## 2 Materials and methods

### 2.1 Study participants

A consecutive series of patients diagnosed with perianal abscess and hospitalized at our institution from August 2019 to March 2025 was retrospectively reviewed. Inclusion criteria comprised: (a) age ≥18 years; (b) diagnosis consistent with the 2016 American Society of Colon and Rectal Surgeons (ASCRS) clinical practice guidelines for perianal abscess (21); (c) absence of MRI contraindications and completion of perianal MRI. Exclusion criteria included: (a) recurrent abscess or postoperative follow-up MRI; (b) preoperative MRI performed without subsequent surgical intervention; (c) missing or negative postoperative pathogen culture results; (d) poor image quality affecting diagnostic accuracy.

**Figure 1:**
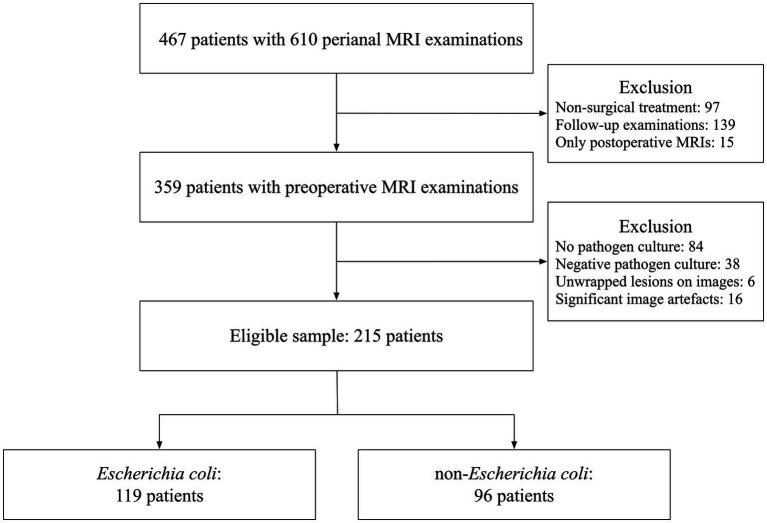
Flowchart of patient selection.

Among 467 patients (610 MRI examinations), 359 underwent preoperative MRI. After excluding 84 patients without pathogen cultures, 38 with negative cultures, 6 with incomplete lesion coverage on imaging, and 16 with significant artifacts, 215 patients were included. These were categorized into two groups based on microbiological profiles: Group 1 (*Escherichia coli*, *n* = 119) and Group 2 (non-*Escherichia coli* pathogens, including single or mixed organisms, *n* = 96), as depicted in Figure 1.

### 2.2 Microbiological analysis

**Figure 2:**
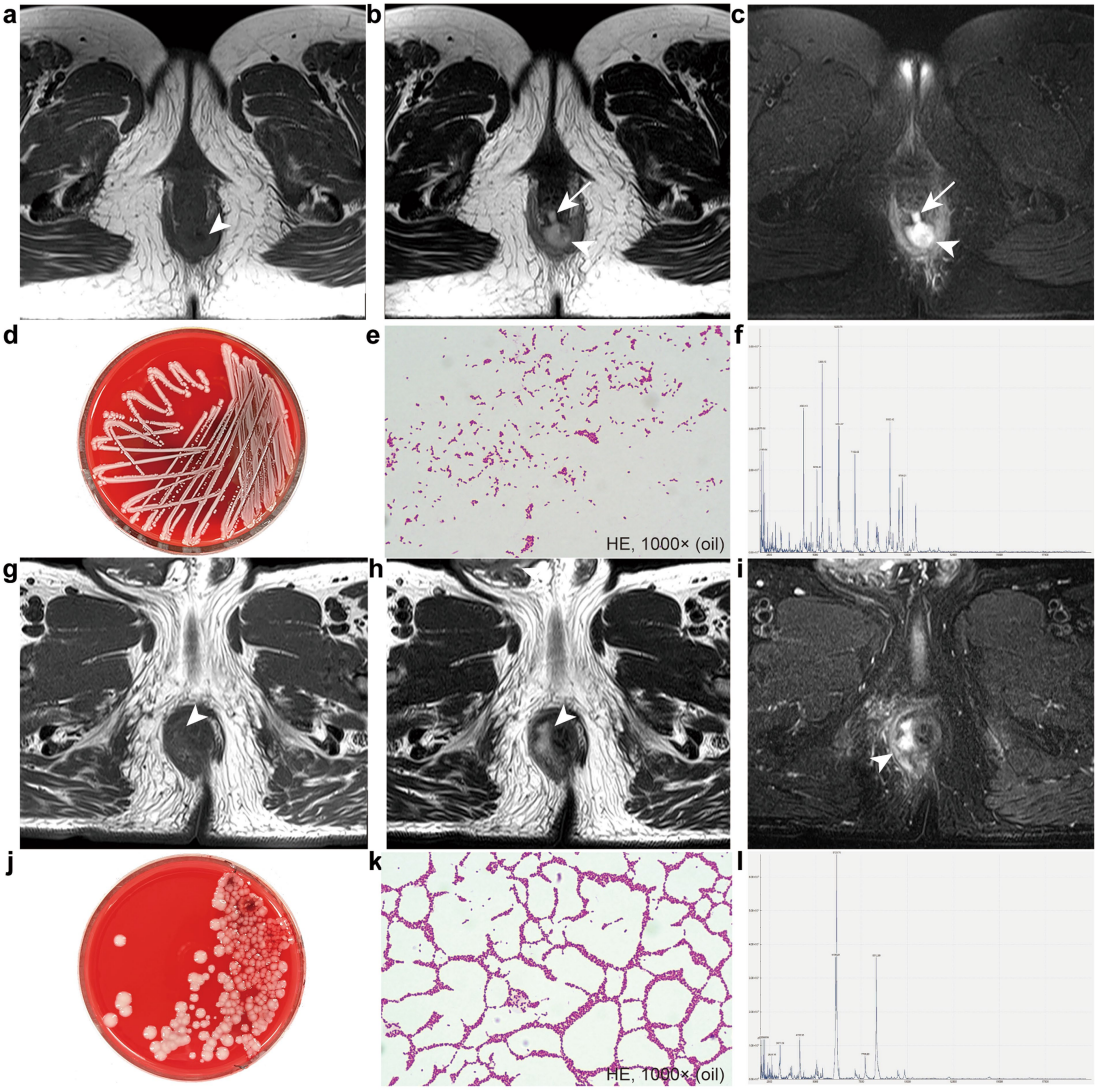
MRI findings and pathogen distribution in patients with perianal abscesses. **(a–f)** *Escherichia coli* infralevator abscess: Oblique axial T1WI **(a)**, T2WI **(b)**, FS-T2WI **(c)** show posterior anal canal abscess (arrowhead) with fistulous tract (arrow). Microbiology: Blood agar culture **(d)**, Gram stain [×1,000 oil-immersion, **(e)**] MALDI-TOF mass spectrometry identification **(f)**. **(g–l)** *Klebsiella pneumoniae* intersphincteric abscess: Corresponding MRI **(g–i)** localizes lesion (arrowhead), with matched microbiology workflow **(j-l)**.

Following surgical drainage of perianal abscesses, purulent specimens (2–5 mL) were aseptically aspirated using sterile syringes and inoculated onto blood agar and MacConkey agar plates (YiHua Biotechnology, China) for incubation at 35 °C with 5–10% CO₂ (18–24 h). Bacterial isolates were Gram-stained for microscopic analysis (including oil-immersion examination) and subsequently identified via matrix-assisted laser desorption/ionization time-of-flight (MALDI-TOF) mass spectrometry (EXS1600 system, Zybio, China), where microbial protein spectra were matched against the manufacturer’s validated in-house database for species identification, as illustrated in Figure 2.

### 2.3 MRI image acquisition

All examinations were performed on a 3.0 T MR scanner (Vantage Titan, Canon Medical Systems Corporation, Japan) using a 16-channel phased-array surface coil. Patients were positioned head-first supine with the magnetic isocenter localized at the pubic symphysis. No bowel preparation or antispasmodic agents were administered. Oblique axial and coronal planes were systematically oriented perpendicular and parallel to the anal canal long axis, respectively. A standardized protocol was implemented for all participants, and detailed sequence scanning parameters were listed below. T1-weighted imaging (T1WI): repetition time (TR) = 480 ms, echo time (TE) = 10 ms; T2-weighted imaging (T2WI): repetition time (TR) = 4,789 ms, echo time (TE) = 80 ms; fat-suppressed T2WI (FS-T2WI): repetition time (TR) = 6,641 ms, echo time (TE) = 80 ms, fat suppression = spectral attenuated inversion recovery (SPAIR). Other identical parameters for the three sequences: two-dimensional fast spin echo (2D FSE) sequence, oblique axial plane, slice thickness = 4 mm, intersection gap = 0.4 mm, flip angle (FA) = 90/160 degrees, bandwidth (BW) = 244.1 Hz/pixel, field of view (FOV) = 250 mm × 200 mm, acquisition matrix = 256 × 288, number of slices = 26. To minimize acquisition variability across the study period, this standardized protocol and scanner configuration were strictly maintained for all patients without parameter alterations.

### 2.4 Radiomics analysis

#### 2.4.1 Image preprocessing and regions of interest (ROIs) segmentation

T1WI and FS-T2WI were rigidly registered to T2WI using the ANTs plugin in 3D-Slicer (version 5.4.0).^1^ A radiologist with >10 years of subspecialty experience in perianal MRI diagnostics manually delineated lesions on T2WI, blinded to microbiological findings. Annotations were cross-verified against T1WI and FS-T2WI, focusing on the largest lesion in multi-lesion cases.

**Figure 3:**
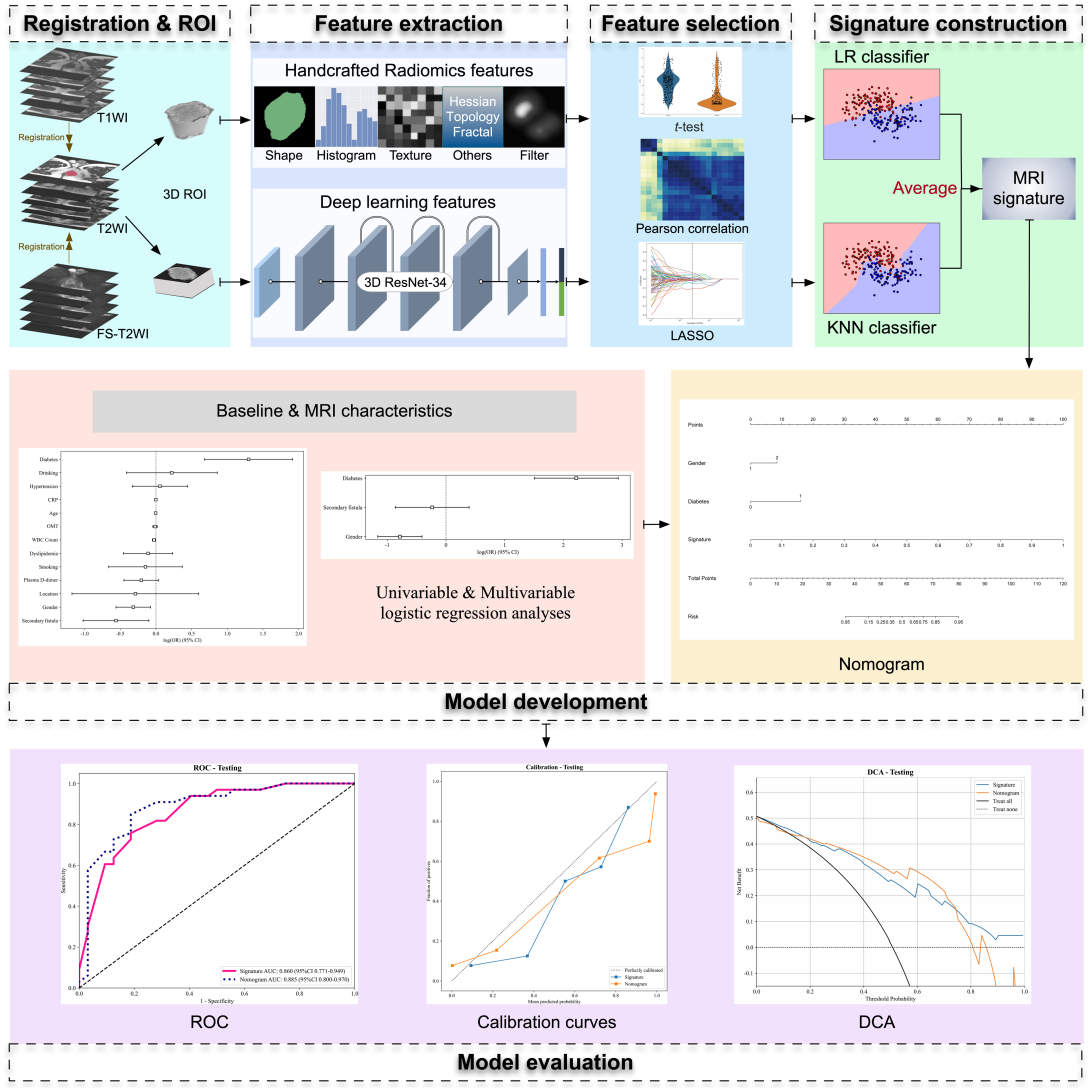
Workflow of the study design.

ROIs were delineated by continuously contouring along the lesion margins to encompass the abscess wall, while rigorously excluding adjacent fat, gas, and osseous structures, ultimately generating irregular three-dimensional (3D) volumetric masks. These T2WI-based ROIs were directly applied to registered T1WI/FS-T2WI. The largest cross-sectional lesion area was cropped to create 3D ROIs containing exclusively lesion-affected regions for deep learning input, as shown in Figure 3.

#### 2.4.2 Feature extraction and selection

Traditional handcrafted radiomic features were extracted using Pyradiomics,^2^ encompassing original images and derived image types generated through wavelet transforms, Laplacian of Gaussian (LoG) filtering, 3D local binary patterns (LBP3D), and nonlinear intensity transformations (exponential, square, square-root, logarithmic) alongside gradient-based filters. Image preprocessing included Z-score normalization based on voxel intensity values across the entire image volume, followed by scaling (factor = 100) and shifting (voxel array shift = 300) to ensure positive intensities. Subsequently, images were isotropically resampled to 2 × 2 × 2 mm^3^ voxels, and histogram discretization was performed with a fixed bin width of 5. Extracted features spanned morphological characteristics, first-order statistical metrics, and high-order texture features derived from gray-level co-occurrence matrices (GLCM), gray-level run-length matrices (GLRLM), gray-level size zone matrices (GLSZM), neighboring gray-tone difference matrices (NGTDM), and gray-level dependence matrices (GLDM). Additional features included Hessian matrix, topological radiomics, and fractal features. Deep learning features were extracted using a ResNet-34 network pretrained on ImageNet. To preserve the original spatial and anatomical fidelity of the volumetric MRI data, no artificial data augmentation was applied. The model was configured with a 3D ROI size of 128 × 128 × 96 voxels, trained for a maximum of 200 epochs with a batch size of 4, an initial learning rate of 0.001, and optimized using the Adam algorithm. During model development, a strict hold-out validation strategy was employed. The model was evaluated on an independent validation set at the end of each epoch. The optimal model weights yielding the highest validation accuracy were automatically saved and selected for subsequent feature extraction, effectively preventing overfitting. Handcrafted and deep learning features were aggregated across T1WI, T2WI, and FS-T2WI sequences.

**Figure 4:**
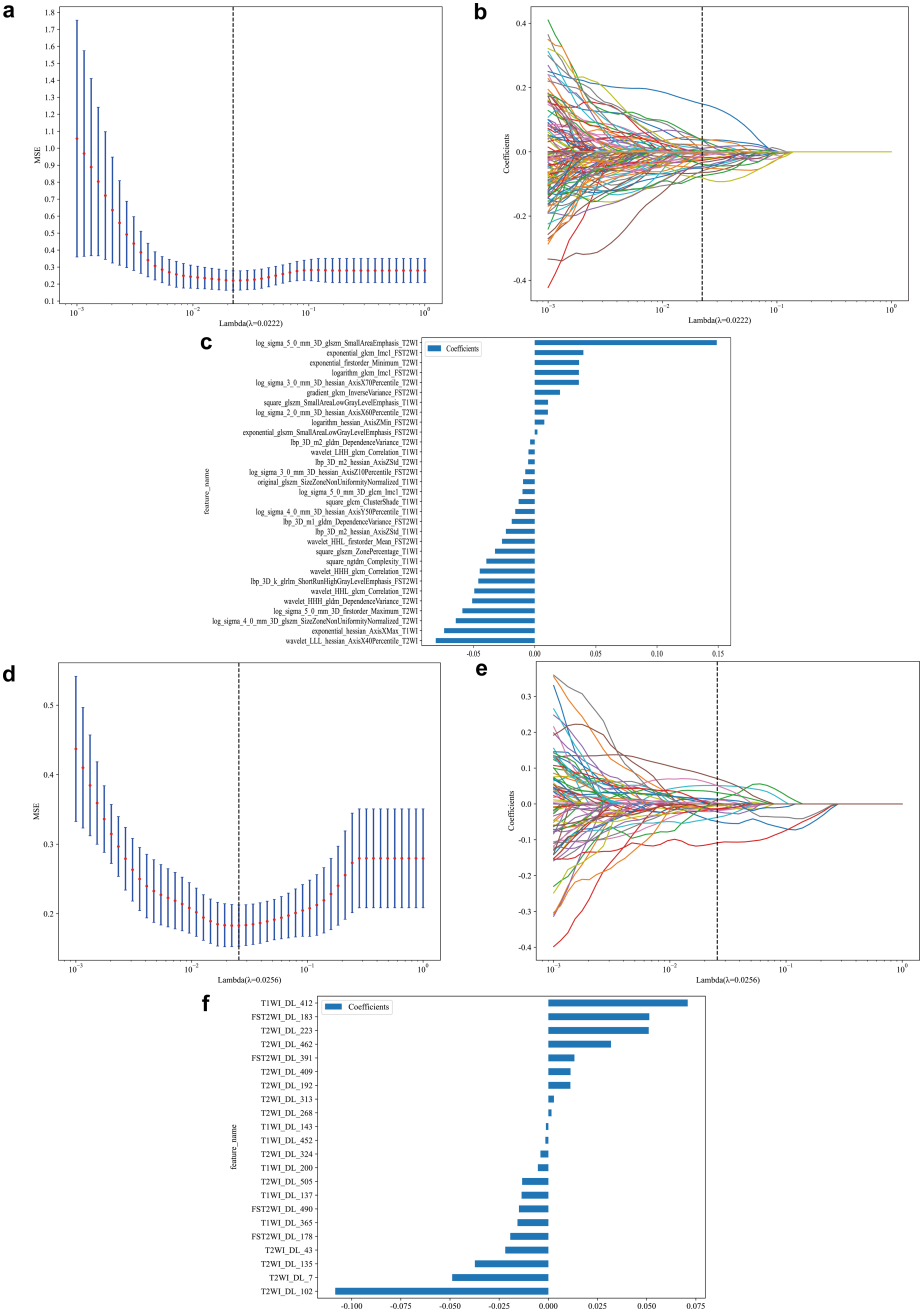
Feature selection using LASSO regression. Handcrafted radiomics **(a–c)** and deep learning-based **(d–f)** feature selection: Optimal *λ* values selected by 10-fold cross-validation with minimal MSE [vertical dashed lines, **(a,d)**]; LASSO coefficient profiles across λ values with vertical dashed lines indicating feature count at optimal λ **(b,e)**; retained feature weights **(c,f)**.

To strictly prevent data leakage, both the initial Z-score normalization (mean = 0, standard deviation = 1) of all extracted features and the subsequent feature selection steps were performed exclusively within the training set. Feature selection for both modalities involved a sequential pipeline: (a) univariate *t*-tests with a significance threshold of *p* < 0.05; (b) removal of highly correlated features with Pearson correlation coefficient *r* > 0.9; and (c) least absolute shrinkage and selection operator (LASSO) regression. The optimal regularization parameter (*λ*) for LASSO was selected through 10-fold cross-validation to minimize the mean squared error (MSE). Features with non-zero coefficients exceeding 1 × 10^−6^ at the optimal λ were retained to construct the feature signature, which was subsequently applied to the testing set, as illustrated in Figure 4. Baseline and MRI characteristics were initially screened via univariate logistic regression, with retained features further refined through multivariable logistic regression to identify final predictors.

#### 2.4.3 MRI signature construction

Selected handcrafted features were input into a logistic regression (LR) model to generate prediction probabilities, while deep learning features were processed using a k-nearest neighbors (KNN) classifier. KNN was specifically selected as its distance-based, non-parametric nature effectively leverages the spatial clustering of abstract deep learning features in the latent space. The MRI signature was derived through an averaging ensemble method, defined as the arithmetic mean of predictions from both models, as illustrated in Figure 3.

#### 2.4.4 Development and assessment of models

The MRI signature and selected baseline/MRI characteristics were integrated into a multivariable logistic regression model for pathogen classification in perianal abscesses, visualized as a nomogram. Model performance was evaluated using receiver operating characteristic (ROC) curve analysis for both the standalone MRI signature and the nomogram-integrated model. The optimal classification threshold was determined by maximizing the Youden index, with performance metrics including the area under the curve (AUC), accuracy, sensitivity, specificity, positive predictive value (PPV), and negative predictive value (NPV). Calibration curves assessed agreement between predicted probabilities and observed outcomes, while decision curve analysis (DCA) was applied to evaluate the clinical utility of both models.

### 2.5 Statistical analysis

Continuous variables were expressed as mean ± standard deviation (SD) or median with interquartile range (IQR) and compared using the independent *t*-test or Mann–Whitney *U* test, as appropriate. Categorical variables were described as counts and percentages, analyzed by Fisher’s exact test or chi-square test. Feature selection was performed via *t*-tests, Pearson correlation analysis, LASSO regression, and univariate/multivariable logistic regression. Model performance was evaluated using ROC curves, reporting AUC, accuracy, sensitivity, specificity, PPV and NPV. All analyses were conducted using Statistical Package for the Social Sciences (SPSS) software (version 26.0; IBM, Chicago, USA) and Python (version 3.12).^3^ A two-tailed *p* value of less than 0.05 was considered statistically significant.

## 3 Results

### 3.1 Baseline and MRI characteristics

Pathogen analysis revealed dual-pathogen infections in 22 patients, with the remaining cases involving single pathogens. The predominant pathogens in perianal abscesses were *Escherichia coli* (136 strains, 57.63%) and *Klebsiella pneumoniae* (56 strains, 23.73%), while other species were minimally represented (Table 1). Representative MRI findings and corresponding culture-confirmed cases of *Escherichia coli* and *Klebsiella pneumoniae* are demonstrated in Figure 2.

**Table 1:** Distribution of pathogenic bacteria in perianal abscesses.

Pathogen	Number of strains	Proportion (%)
Gram-negative bacteria	213	90.25
*Escherichia coli*	136	57.63
*Klebsiella pneumoniae*	56	23.73
*Proteus mirabilis*	7	2.97
*Edwardsiella tarda*	3	1.27
*Morganella morganii*	3	1.27
*Enterobacter hormaechei*	2	0.85
*Citrobacter freundii*	2	0.85
*Citrobacter farmeri*	1	0.42
*Enterobacter aerogenes*	1	0.42
*Acinetobacter baumannii*	1	0.42
*Proteus vulgaris*	1	0.42
Gram-positive bacteria	22	9.33
*Streptococcus anginosus*	8	3.39
*Streptococcus constellatus*	4	1.70
*Streptococcus agalactiae*	4	1.70
*Enterococcus faecalis*	3	1.27
*Staphylococcus aureus*	2	0.85
*Enterococcus avium*	1	0.42
Fungus	1	0.42
*Candida albicans*	1	0.42

A total of 215 samples were randomly partitioned into training (*n* = 150) and testing (*n* = 65) sets using a 7:3 ratio. All 13 baseline and MRI characteristics exhibited comparable distributions between training and testing sets (*p* > 0.05 for all; Table 2).

**Table 2:** Baseline and MRI characteristics in training and testing sets.

Characteristic	All (*n* = 215)	Training set (*n* = 150)	Testing set (*n* = 65)	*p*-value
Age, years	44.11 ± 13.97	44.52 ± 14.69	43.17 ± 12.18	0.803
OMT, days	7.32 ± 5.02	7.25 ± 5.06	7.46 ± 4.97	0.519
WBC Count, ×10^9^/L	11.26 ± 3.25	11.32 ± 3.38	11.11 ± 2.95	0.837
CRP, mg/L	28.06 ± 43.18	29.45 ± 47.89	24.86 ± 29.63	0.507
Plasma D-dimer, μg/mL	0.99 ± 0.50	1.00 ± 0.52	0.98 ± 0.47	0.855
Gender, *n* (%)				0.367
Male	198 (92.09)	136 (90.67)	62 (95.38)	
Female	17 (7.91)	14 (9.33)	3 (4.62)	
Smoking, *n* (%)				0.727
No	154 (71.63)	109 (72.67)	45 (69.23)	
Yes	61 (28.37)	41 (27.33)	20 (30.77)	
Diabetes, *n* (%)				0.729
No	157 (73.02)	108 (72.00)	49 (75.38)	
Yes	58 (26.98)	42 (28.00)	16 (24.62)	
Drinking, *n* (%)				0.582
No	179 (83.26)	123 (82.00)	56 (86.15)	
Yes	36 (16.74)	27 (18.00)	9 (13.85)	
Dyslipidemia, *n* (%)				0.322
No	90 (41.86)	59 (39.33)	31 (47.69)	
Yes	125 (58.14)	91 (60.67)	34 (52.31)	
Hypertension, *n* (%)				0.177
No	119 (55.35)	78 (52.00)	41 (63.08)	
Yes	96 (44.65)	72 (48.00)	24 (36.92)	
Location, *n* (%)				1.000
Infralevator	195 (90.70)	136 (90.67)	59 (90.77)	
Supra/Translevator	20 (9.30)	14 (9.33)	6 (9.23)	
Secondary fistula, *n* (%)				0.809
No	138 (64.19)	95 (63.33)	43 (66.15)	
Yes	77 (35.81)	55 (36.67)	22 (33.85)	

OMT, onset-to-MRI time (time interval from symptom onset to MRI acquisition); WBC, white blood cell; CRP, C-reactive protein.

Univariate logistic regression identified gender, diabetes, and secondary fistula as significant predictors (*p* < 0.05), with odds ratios (ORs) of 0.728, 3.667, and 0.571, respectively. Multivariable analysis retained gender (OR = 0.456) and diabetes (OR = 9.207) as independent clinical predictors (Table 3).

**Table 3:** Univariable and multivariable analyses of baseline and MRI characteristics.

Characteristic	Univariable analysis		Multivariable analysis	
	OR (95% CI)	*p*-value	OR (95% CI)	*p*-value
Age	0.995 (0.989–1.001)	0.168		
OMT	0.988 (0.958–1.019)	0.519		
WBC count	0.974 (0.951–0.996)	0.058		
CRP	0.999 (0.994–1.004)	0.710		
Plasma D-dimer	0.815 (0.638–1.040)	0.167		
Gender	0.728 (0.571–0.929)	**0.032**	0.456 (0.313–0.664)	**0.001**
Smoking	0.864 (0.516–1.446)	0.640		
Diabetes	3.667 (1.976–6.807)	**0.001**	9.207 (4.509–18.803)	**<0.001**
Drinking	1.250 (0.661–2.363)	0.565		
Dyslipidemia	0.896 (0.634–1.265)	0.600		
Hypertension	1.057 (0.717–1.557)	0.814		
Location	0.750 (0.309–1.824)	0.594		
Secondary fistula	0.571 (0.360–0.906)	**0.046**	0.791 (0.421–1.484)	0.539

OMT, onset-to-MRI time; WBC, white blood cell; CRP, C-reactive protein; OR, odds ratio; CI, confidence interval. Bold indicates *p* < 0.05.

### 3.2 Feature extraction and selection

The feature selection process yielded 31 optimal handcrafted radiomics features from an initial pool of 8,364, comprising 3 first-order statistics, 6 GLSZM, 8 GLCM, 3 GLDM, 1 GLRLM, 1 NGTDM, and 9 Hessian matrix-based features. These features demonstrated sequence-specific predominance, with T2WI contributing the largest subset (13 features, 41.9%), followed by T1WI and FS-T2WI (9 features each). Weight distribution profiling (Figure 4c) revealed differential contributions across feature categories.

For deep learning features, 22 discriminative features were selected from 1,536 candidates, showing a similar T2WI dominance (12 features, 54.5%) compared to T1WI (6 features) and FS-T2WI (4 features), as visualized in Figure 4f. Notably, T2WI features demonstrated comparative predominance in both handcrafted and deep learning paradigms.

### 3.3 Development and performance of models

**Figure 5:**
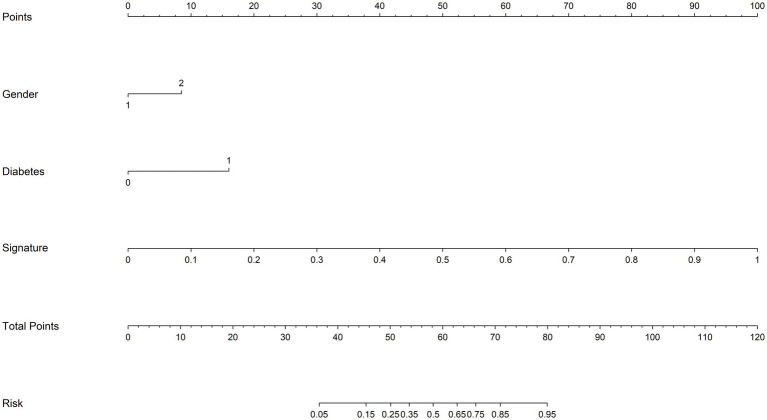
Nomogram combining clinical features and MRI signature.

**Figure 6:**
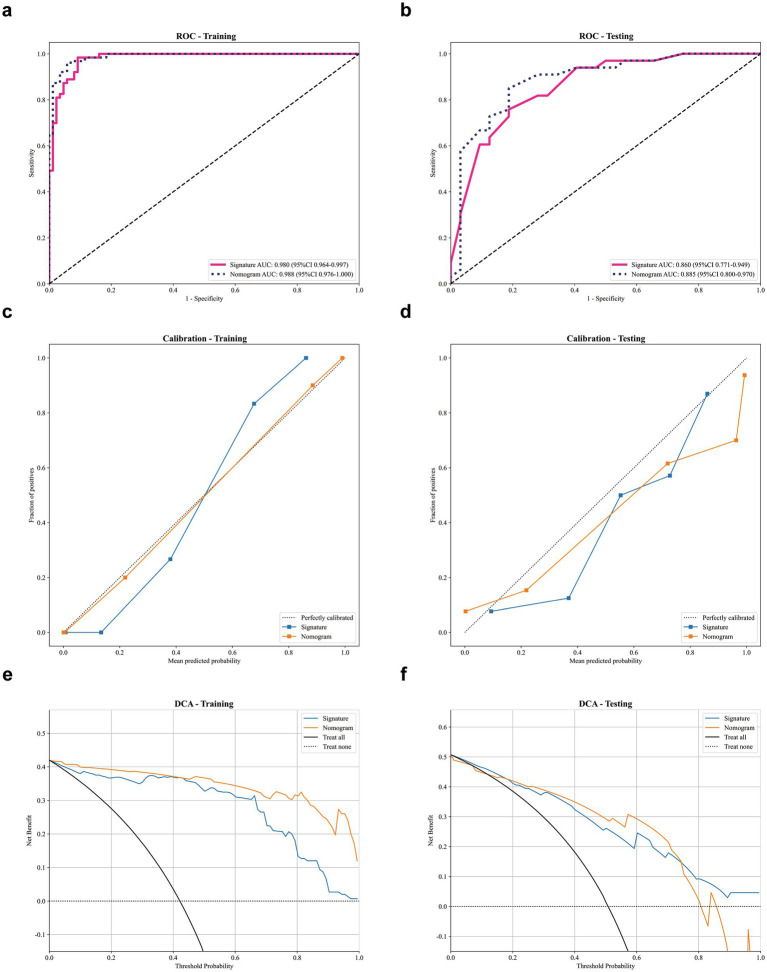
Comprehensive assessment of the predictive model’s performance. **(a,b)** ROC curves in the training and testing sets. **(c,d)** Calibration plots in the training and testing sets. **(e,f)** Decision curve analysis in the training and testing sets.

The integrative nomogram (Figure 5), combining gender and diabetes with the MRI signature, outperformed the MRI signature-alone model in discriminating *Escherichia coli* from non-*Escherichia coli* pathogens. In the testing set, the nomogram achieved superior metrics: AUC 0.885 vs. 0.860, accuracy 0.815 vs. 0.769, sensitivity 0.818 vs. 0.727, and NPV 0.812 vs. 0.743, while maintaining comparable specificity (0.812) and PPV (0.818 vs. 0.800) (Table 4; Supplementary Table S1; Figures 6a,b). DCA in both training and testing sets showed superior net clinical benefit of the Nomogram model over the MRI signature model (Figures 6e,f). Calibration curves further confirmed good agreement between predicted probabilities and actual observations (Figures 6c,d).

**Table 4:** Performance of the MRI signature and nomogram in training and testing sets.

Model	Set	AUC (95% CI)	Accuracy	Sensitivity	Specificity	PPV	NPV
MRI signature	Train	0.980 (0.964–0.997)	0.933	0.968	0.908	0.884	0.975
MRI signature	Test	0.860 (0.771–0.949)	0.769	0.727	0.812	0.800	0.743
Nomogram	Train	0.988 (0.976–0.999)	0.940	0.952	0.931	0.909	0.964
Nomogram	Test	0.885 (0.800–0.969)	0.815	0.818	0.812	0.818	0.812

AUC, area under curve; CI, confidence interval; PPV, positive predictive value; NPV, negative predictive value.

## 4 Discussion

Early identification of causative pathogens is critical for optimizing perioperative management and conservative therapy in perianal abscesses, enabling rational antibiotic use and mitigating antimicrobial resistance. Given the current lack of effective preoperative methods for microbial classification, we developed a hybrid model based on preoperative multi-sequence MRI. This model integrates traditional radiomics and deep learning features via an averaging ensemble method, combined with two clinical baseline characteristics (gender and diabetes), to effectively discriminate *Escherichia coli*—the predominant pathogen in perianal abscesses—achieving an AUC of 0.885 and demonstrating promising clinical utility.

In our study, *Escherichia coli* and *Klebsiella pneumoniae* constituted the two predominant pathogens (57.63 and 23.73%, respectively), aligning with epidemiological patterns reported in adults (9) and pediatric populations (22). Given this distribution, we adopted a binary grouping strategy (*Escherichia coli* vs. non-*Escherichia coli*) for model development. This pragmatic approach was necessary to prevent severe class imbalance and model overfitting that would inevitably occur if the non-*Escherichia coli* cohort—comprising diverse single and mixed organisms—were further subdivided. We acknowledge that this grouping introduces microbiological heterogeneity, as mixed organisms and Gram-positive skin flora may exhibit varying inflammatory patterns, pus viscosities, and tissue edema on MRI. Consequently, our model’s interpretation focuses on identifying the specific, dominant imaging signature of *Escherichia coli* against a heterogeneous background. This ensures robust identification of the predominant pathogen to guide initial Gram-negative targeted therapy; however, it also means that while a ‘non-*Escherichia coli*’ prediction successfully rules out the primary pathogen, it cannot pinpoint the exact alternative bacterial species (e.g., distinguishing between *Klebsiella* or *Staphylococcus*). Established risk factors for perianal abscesses, including smoking, obesity, diabetes, and inflammatory bowel disease, were consistent with prior literature (4–7, 23). Notably, both gender and diabetes demonstrated statistical significance in univariate analysis and retained independent predictive value in multivariate logistic regression, supporting their utility for pathogen stratification and subsequent integration as clinical covariates in the final model. Although secondary fistulas were excluded during multivariate feature selection, they remain a critical radiological marker in preoperative MRI evaluation, profoundly influencing surgical strategy formulation and long-term prognosis.

MRI is routinely utilized for preoperative diagnosis, surgical planning, and complication monitoring in perianal abscess management (8, 13, 14), with three-dimensional reconstructions from MRI data providing anatomically precise visualization of abscess morphology (24). Beyond structural characterization, dynamic contrast-enhanced MRI (DCE-MRI) enables functional assessment of fistula tract activity (25). In a novel translational extension, we pioneered MRI-based prediction of pathogen categories in perianal abscesses. Analysis of final selected features revealed T2WI as the dominant sequence contributor, accounting for 41.94 and 54.55% of handcrafted radiomic and deep learning features respectively, with T2WI also yielding the highest feature importance weights. This T2WI predominance likely reflects its superior contrast resolution for free water signals and inflammatory edema—non-fat-suppressed protocols enhance boundary delineation between abscess cavities (hyperintense on T2WI) and surrounding fibrotic tissue (hypointense). By leveraging the inherent contrast of these non-contrast sequences (T1WI, T2WI, and FS-T2WI), contrast-enhanced protocols were omitted, thereby developing a completely “contrast-free” model that mitigates gadolinium-related risks, expands applicability to patients with contraindications like renal insufficiency, and enhances cost-effectiveness for clinical implementation.

The ResNet-34 deep learning framework was employed for feature extraction, an architecture extensively validated in radiomics research (26, 27). Its residual block design mitigates gradient vanishing in deep network training, while the streamlined architecture proves advantageous for small-to-medium medical imaging datasets, efficiently capturing localized ROI features such as intralesional heterogeneity and abscess wall variations. Transfer learning-derived features (ImageNet pre-training) synergized with traditional handcrafted radiomics, demonstrating robust feasibility for lesion classification. Prior studies highlight deep learning’s emerging potential in perianal diseases—for instance, MRI-based models outperformed human assessments in differentiating perianal fistulizing Crohn’s disease from cryptoglandular fistulas (28), and multimodal fusion algorithms improved segmentation accuracy and classification of perianal abscesses and fistulas (29). We advocate broader adoption of deep learning to automate structured reporting of perianal abscesses and anal fistulas, thereby enhancing diagnostic accuracy and workflow efficiency in pelvic imaging.

Beyond diagnostic accuracy, the practical clinical utility of this hybrid model lies in its potential to seamlessly integrate into and optimize routine workflows. Currently, the 48- to 72-h delay in obtaining traditional pus culture results in clinical practice forces clinicians to rely heavily on broad-spectrum empirical antibiotics, which cover both enteric (Gram-negative) and skin-derived (Gram-positive) flora. By acting as a ‘virtual preoperative biopsy,’ our model provides an immediate, high-probability prediction of *Escherichia coli*. This allows clinicians to confidently narrow the antimicrobial spectrum to target enteric bacteria early in the treatment course, directly supporting antimicrobial stewardship and mitigating the selective pressure that drives resistance. Furthermore, real-world clinical implementation is highly feasible. The model utilizes standard non-contrast MRI sequences already acquired during routine care. Post-acquisition, the entire pipeline—including manual ROI segmentation and automated feature extraction and classification—can be completed within minutes. This rapid turnaround time ensures that microbiology-guided precision strategies can be formulated preoperatively without delaying surgical intervention.

Despite the encouraging results, this study has several limitations. First, the absence of external validation is a major limitation of this work. Given the single-center retrospective design and relatively small sample size, the generalizability and robustness of the proposed hybrid model across different hospital settings, diverse patient populations, and varying MRI scanner protocols remain uncertain. To address this limitation and facilitate clinical translation, we are currently planning a prospective, multicenter study to externally validate and fine-tune the model’s performance in real-world clinical workflows. Second, the discrepancy between the training and testing AUCs suggests a certain degree of overfitting, which is a common challenge when handling high-dimensional radiomics and deep learning features with a relatively limited sample size. To mitigate this risk and enhance model robustness, we implemented strict data isolation to prevent data leakage, applied rigorous dimensionality reduction (univariate *t*-tests, Pearson correlation, and LASSO with 10-fold cross-validation), and utilized a relatively simple linear classifier (logistic regression) for the final nomogram. Nevertheless, future studies with larger sample sizes will be crucial to further stabilize the model and minimize overfitting. Third, preoperative low-dose antibiotic administration in a subset of patients could have affected microbial culture outcomes, potentially confounding pathogen distribution patterns. Fourth, the limited study size precluded stratification by variable intervals between symptom onset and MRI examination, hindering phase-restricted analyses of abscess formation that might strengthen imaging-pathogen correlations. Fifth, ROI segmentation was performed manually by a single radiologist. Although the radiologist had over 10 years of subspecialty experience and cross-verified annotations across multiple MRI sequences to ensure baseline accuracy, the lack of formal interobserver and intraobserver reproducibility analysis (such as Intraclass Correlation Coefficient, ICC) introduces potential subjective variability. Future studies should incorporate multiple annotators or automated deep-learning-based segmentation algorithms to further validate the robustness and reproducibility of the extracted features.

## 5 Conclusion

This study presents an innovative multimodal framework integrating radiomics and deep learning features derived from preoperative MRI to enable noninvasive identification of *Escherichia coli*—the predominant pathogen in perianal abscesses. The model’s robust performance underscores its potential to advance precision-guided therapeutic strategies, offering a pathway to transition from empirical antibiotic therapies to microbiologically informed decision-making in both conservative management and perioperative care.

## Data Availability

The original contributions presented in the study are included in the article/Supplementary material, further inquiries can be directed to the corresponding author.

## Footnotes

1
https://www.slicer.org

2
http://pyradiomics.readthedocs.io

3
https://www.python.org
