# Supplementary material for: MRI-based radiomics-deep learning model for preoperative pathogen prediction in perianal abscesses
Source: Front Med (Lausanne). 2026 Jun 17;13:1865284. doi: 10.3389/fmed.2026.1865284 (PMC13318606; doi:10.3389/fmed.2026.1865284)
Supplement: Supplementary file 1 [file Table_1.DOCX]

**Supplementary document**

**Supplementary Table 1** Performance of the MRI signature and nomogram in training and testing sets

| Model | Set | AUC  (95% CI) | Accuracy | Sensitivity  (95% CI) | Specificity  (95% CI) | PPV | NPV |
| --- | --- | --- | --- | --- | --- | --- | --- |
| MRI signature | train | 0.980  (0.964 - 0.997) | 0.933 | 0.968  (0.913 - 0.993) | 0.908  (0.825 - 0.961) | 0.884 | 0.975 |
| MRI signature | test | 0.860  (0.771 - 0.949) | 0.769 | 0.727  (0.617 - 0.874) | 0.812  (0.692 - 0.877) | 0.800 | 0.743 |
| Nomogram | train | 0.988  (0.976 - 0.999) | 0.940 | 0.952  (0.885 - 0.985) | 0.931  (0.843 - 0.969) | 0.909 | 0.964 |
| Nomogram | test | 0.885  (0.800 - 0.969) | 0.815 | 0.818  (0.703 - 0.928) | 0.812  (0.692 - 0.877) | 0.818 | 0.812 |

AUC = area under curve; CI = confidence interval; PPV = positive predictive value; NPV = negative predictive value
